# Genetic code expansion as a tool to study regulatory processes of transcription

**DOI:** 10.3389/fchem.2014.00007

**Published:** 2014-02-25

**Authors:** Moritz J. Schmidt, Daniel Summerer

**Affiliations:** Department of Chemistry, Zukunftskolleg and Konstanz Research School Chemical Biology, University of KonstanzKonstanz, Germany

**Keywords:** genetic code expansion, non-canonical amino acids, transcription, epigenetics, protein, nucleic acid interactions

## Abstract

The expansion of the genetic code with non-canonical amino acids (ncAA) enables the chemical and biophysical properties of proteins to be tailored, inside cells, with a previously unattainable level of precision. A wide range of ncAA with functions not found in canonical amino acids have been genetically encoded in recent years and have delivered insights into biological processes that would be difficult to access with traditional approaches of molecular biology. A major field for the development and application of novel ncAA-functions has been transcription and its regulation. This is particularly attractive, since advanced DNA sequencing- and proteomics-techniques continue to deliver vast information on these processes on a global level, but complementing methodologies to study them on a detailed, molecular level and in living cells have been comparably scarce. In a growing number of studies, genetic code expansion has now been applied to precisely control the chemical properties of transcription factors, RNA polymerases and histones, and this has enabled new insights into their interactions, conformational changes, cellular localizations and the functional roles of posttranslational modifications.

## Introduction

Genetic code expansion has become an important tool to study biological processes both *in vitro* and in living cells. This approach relies on heterologous pairs of aminoacyl-tRNA-synthetases (aaRS) and tRNAs that enable the co-translational incorporation of non-canonical amino acids (ncAA) in a host organism, in response to unique non-sense codons such as the amber codon, UAG (Liu and Schultz, 2010). The selectivity of the incorporation process is tightly controlled at several steps, in order to maintain the integrity of the information transfer from the gene to the encoded protein: the heterologous aaRS and tRNA build a functional pair, i.e., the aaRS can aminoacylate the tRNA and the aminoacylated tRNA is compatible with the downstream translation components of the host, such as elongation factors and the ribosome. However, it is also orthogonal, i.e. the tRNA is not a substrate of the hosts aaRS and the hosts tRNAs are not substrates of the heterologous aaRS. Moreover, the ncAA itself must not be a substrate for host aaRS and has to be cell-permeable, non-toxic and metabolically stable.

The initial discoveries of several orthogonal tRNA/aaRS pairs and their extensive re-engineering for the selective processing of novel ncAA have provided a comprehensive toolbox with diverse functions for biological studies. Furthermore, methodological advancements, such as the development of tRNA/aaRS expression strategies for an increasing range of organisms (Young et al., 2009; Wang et al., 2010a; Greiss and Chin, 2011; Bianco et al., 2012; Parrish et al., 2012; Kang et al., 2013; Li et al., 2013), ribosome engineering (Wang et al., 2007b; Neumann et al., 2010) and the development of strains with improved non-sense codon suppression efficiencies (Ryden and Isaksson, 1984; Mukai et al., 2010; Johnson et al., 2011; Lajoie et al., 2013; Wu et al., 2013) now enable the efficient, co-translational incorporation of ncAA in a wide range of (multicellular) organisms, also including multiple different ncAA in response to individual codons. For a detailed introduction into this topic, we refer to a recent excellent review (Liu and Schultz, 2010).

## Genetically encoded chemistries for studying regulatory processes of transcription

The introduction of ncAA with novel chemical or biophysical functions by this strategy is not only a particularly simple way to chemically modify proteins for *in vitro* studies, but also allows to study proteins directly in living cells, with minimal perturbation of their structure and their natural environment. A research field that has especially benefitted from these advantages is transcription and its regulation. Here, genetic code expansion has a considerable potential to even out the current imbalance between the wide availability and massive information output of discovery-oriented screening techniques on one side and the limited availability of methods for studying transcriptional mechanisms at the detailed molecular level on the other side. In particular, the diverse assay formats utilizing high throughput sequencing have provided comprehensive knowledge of the general transcriptional activity of the genome (Djebali et al., 2012) and its association state with transcription factors, chromatin remodeling complexes or RNA polymerase components (Bernstein et al., 2012; Neph et al., 2012). Moreover, chromatin accessibility (Thurman et al., 2012) and genomic distributions of distinct posttranslational modifications (PTM) in histones have been precisely mapped (Bernstein et al., 2012). Finally, the recent discoveries of new epigenetic DNA modifications and their associated proteins (Munzel et al., 2011; Song et al., 2012) as well as of new histone PTMs with unknown roles (Du et al., 2011; Tan et al., 2011; Zhang et al., 2011b; Olsen, 2012) have added an additional layer of complexity to the current picture of transcription regulation. This vast amount of findings brings up a multitude of questions, many of which can only be answered by a methodology that allows to more precisely control the chemical properties of the involved proteins by the site-specific installment of ncAA: How can the stoichiometry and topology of transcriptional complexes be accurately assessed in their natural environment? What mechanisms underlie the control of the local concentrations of transcription factors at their genomic target sites? How do individual PTMs in histones or transcription factors control the recognition of their cognate DNA elements or other proteins?

**Figure 1:**
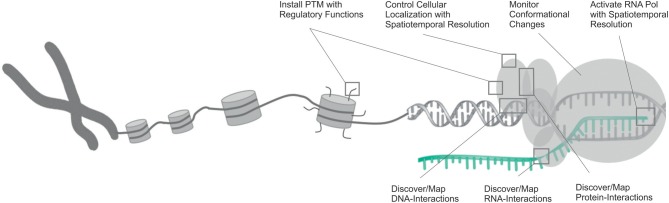
**Overview of the opportunities that have been opened by the use of genetic code expansion in the studying of various aspects of transcription and its regulation**. DNA is shown in gray, RNA in cyan.

**Scheme 1:**
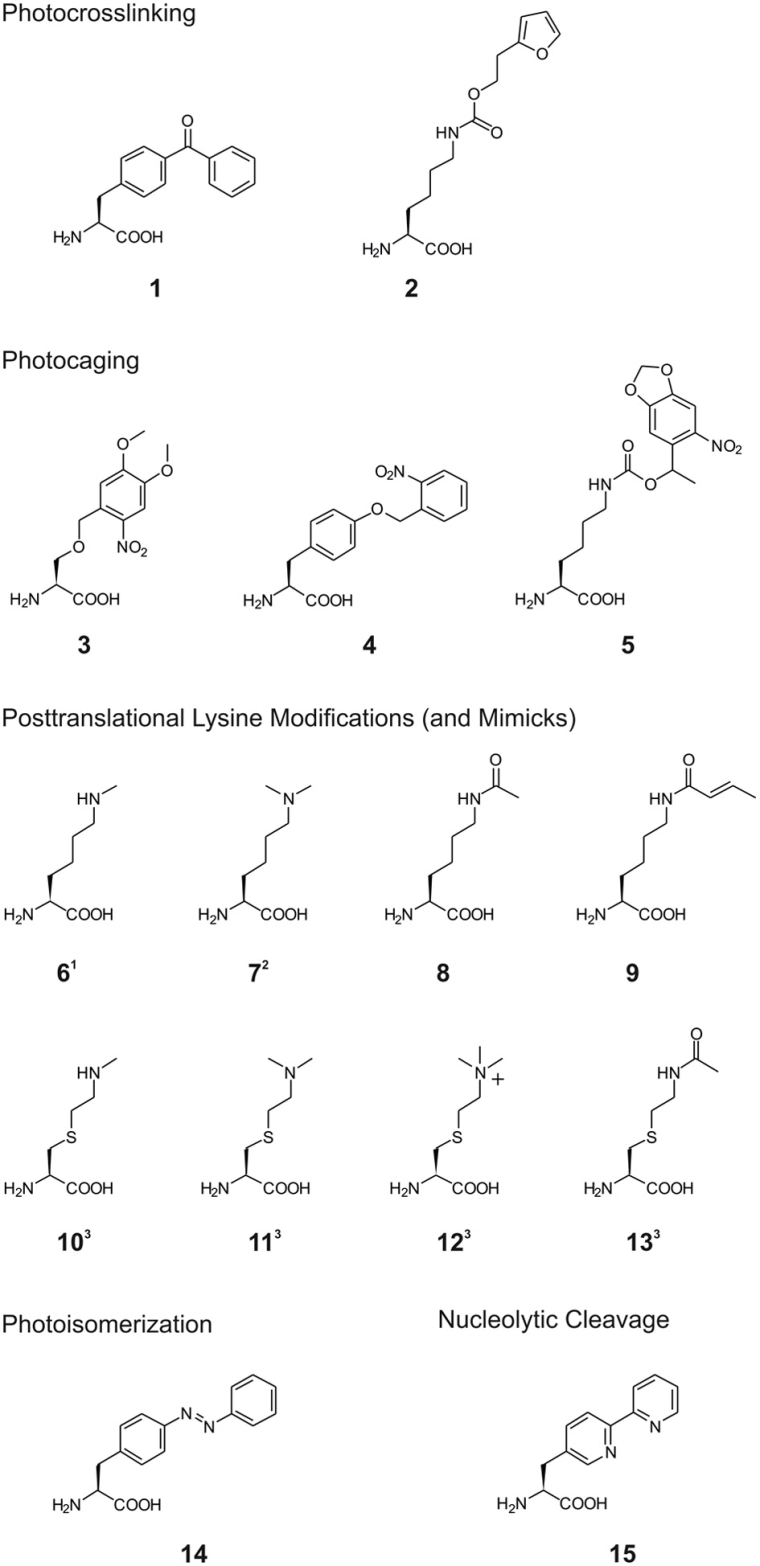
**Structures of non-canonical amino acids (ncAA) used in the reviewed studies**. ^1^: for inorporation of ncAA **6**, different Nε-protected precursors were genetically encoded followed by posttranslational deprotection *in vitro* (see also Scheme 2A). ^2^: for incorporation of ncAA **7**, Nε-Boc-L-lysine was genetically encoded and dimethylation was achieved posttranslationally after deprotection *in vitro* (see also (Scheme 2B). ^3^: ncAA **10**–**13** were incorporated into proteins by incorporation of selenocysteine-derivatives, oxidative elimination to dehydroalanine **20** and subsequent michael additions with thiols (see also (Scheme 2C).

Here, we review recent studies that utilized ncAA and provided first answers in this direction. These demonstrate the considerable potential of genetic code expansion to study fundamental properties of proteins, such as their interactions with other proteins or nucleic acids, transport mechanisms or conformational dynamics (Figure 1). Though generally a wide range of ncAA were used for the above mentioned studies, we focus on photocrosslinkers, photoactivatable ncAA and ncAA with defined PTMs (Scheme 1), since these have had the largest impact in the transcriptional field.

### Photocrosslinking

Key to the understanding of transcriptional complexes is to define their stoichiometry, topology and conformational dynamics by identifying protein interaction partners and mapping their contact surfaces. This can be a difficult task, since the involved interactions may be weak and transient and ideally have to be identified *in vivo*. ncAA with the ability to form covalent bonds to nearby molecules upon irradiation with light have become widely used tools for this purpose. Examples of such photocrosslinking chemistries, already successfully genetically encoded, are based on aryl azides (Chin et al., 2002b), diazirines (Tippmann et al., 2007; Ai et al., 2011; Chou et al., 2011; Lin et al., 2011; Zhang et al., 2011a; Yanagisawa et al., 2012; Chatterjee et al., 2013), benzophenones (Chin et al., 2002a, 2003; Lacey et al., 2013) and furan (Schmidt and Summerer, 2013). Aryl azides, diazirines, and benzophenone form highly reactive intermediates upon irradiation with UV light that subsequently react rather unselectively with nearby molecules. Among these, diazirines and benzophenones are most widely used. While diazirines are the functionality for which the largest variety of structurally distinct ncAA is available and that can be encoded in the largest variety of hosts, including several pathogenic bacteria (Lin et al., 2011; Zhang et al., 2011a), only the benzophenone-containing amino acid **1** (BpA, Scheme 1) has however been used to study transcriptional proteins. In pioneering studies by the Schultz group, ncAA **1** was genetically encoded using a *Methanococcus jannaschii* tRNA^Tyr^/TyrRS pair with an evolved TyrRS mutant in *E. coli* (Chin et al., 2002a) and an aaRS mutant of an *E. coli* tRNA^Tyr^/TyrRS pair (Chin et al., 2003) in eukaryotes. Later, the use of a *Methanosarcina mazei* tRNA^Pyl^/PylRS pair was reported (Lacey et al., 2013). Excited benzophenone preferentially reacts with otherwise unactivated C-H bonds via CH-insertion (Galardy et al., 1973). An advantage is that benzophenone does not photodissociate and that its excited triplet state readily relaxes in the absence of available reaction partners. This reversibility allows repeated excitation and high crosslinking yields (Kauer et al., 1986). A disadvantage, however, is the relatively large size and conformational rigidity of **1**, which could perturb the protein interaction surface under study.

In the only photocrosslinking study so far that utilized ncAA for mapping protein-DNA interactions, **1** was used to characterize the binding of the well-known *E. coli* catabolite activator protein (CAP), a transcriptional activator that regulates catabolite-sensitive operons by binding to DNA in presence of cAMP as allosteric effector (Lee et al., 2009).

**Figure 2:**
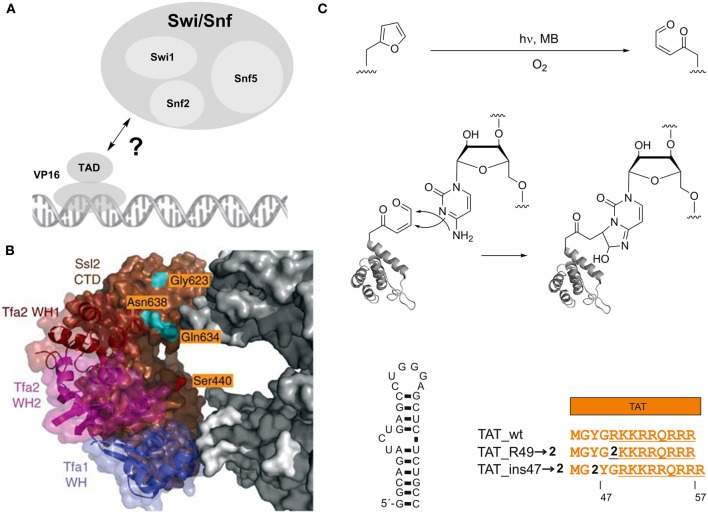
**Photocrosslinking studies with an expanded genetic code**. **(A)** Interaction of the prototypical transcriptional activator VP16 with the nucleosome remodeling complex Swi/Snf. **(B)** Model showing part of the central cleft of RNA polymerase II in the transcription preinitiation complex bound to the general transcrption factors TFIIE and the Ssl2 subunit of TFIIH. Three winged helix (WH) domains of TFIIE are shown in blue, magenta and dark brown, the Ssl2 subunit in light brown. RNA polymerase II is shown in gray, amino acids analyzed in photocrosslinking studies in orange. Adapted by permission from Macmillan Publishers Ltd: Nature Structural and Molecular Biology (Grunberg et al., 2012), copyright 2012. **(C)** Red-light controlled Protein-RNA crosslinking using ncAA **2** bearing a furan moiety. Top: activation of the furan by oxidation with singlet oxygen, resulting in a γ-keto-enal. Middle: Proposed mechanism for the formation of a cyclic adduct between the γ-keto-enal and cytosine. Lower part: Left: HIV-1 TAR. Right: Arginine-rich motif of HIV-1 TAT and incorporation positions of furan-bearing ncAA **2**.

However, **1** has been more widely used for the studying of protein-protein interactions involved in transcription. For example, **1** has provided a deeper understanding of the interplay between transcriptional activators and co-activators. This is of particular interest, since these interactions often are transient and have only moderate affinity, which makes them difficult targets for interaction studies (Melcher, 2000; Mapp and Ansari, 2007; Fuxreiter et al., 2008). Following proof-of-principle experiments to detect and characterize the interaction between Gal4 and its (high affinity) suppressor Gal80 in *S. cerevisiae* (Majmudar et al., 2009a), a series of studies was dedicated to uncover individual binding sites of several transcriptional activators with the co-activator Med15 and the chromatin-modifying coactivator complex Swi/Snf2 (Majmudar et al., 2009b; Krishnamurthy et al., 2011). Swi/Snf2 is a multimeric complex that uses a DNA-stimulated ATPase activity for nucleosome remodeling and plays important roles in tumor suppression (Wilson and Roberts, 2011). It has been proposed to be a direct binding partner of transcriptional activators such as the prototypical activator VP16, but the involved Swi/Snf2 subunits and exact interaction mode *in vivo* has been unclear (Figure 2A). Crosslinking studies using **1** incorporated at different sites in either the N- or C-terminal region of the VP16 transcriptional activation domain (TAD) targeting the three subunits Swi1, Snf2 and Snf5 revealed the ATPase Snf2 as a direct binding partner of the VP16 C-terminal domain. This interaction did also occur with the activator Gal4, suggesting a more general mechanism of activation (Krishnamurthy et al., 2011).

In a further study, the interactions of the *S. cerevisiae* TATA-box binding protein (TBP) were mapped *in vivo* and in isolated transcription pre-initiation complexes (Mohibullah and Hahn, 2008). These revealed direct interactions with the general transcription factor TFIIA and the Spt3 and Spt8 subunits of the multifunctional co-activator SAGA that is extensively involved in histone modification. The insights gained into the interaction with Spt3 by photocrosslinking provided a starting point for a detailed characterization by mutation studies targeting amino acids close to the incorporation sites of **1** that promoted effective crosslinking. Mutations at several sites resulted in a loss of affinity of TBP to SAGA and reduced transcription activation, which demonstrates the value of **1** to identify relevant interactions.

Several studies have focused on the identification of regulatory interaction partners of RNA polymerase and the definition of conformational changes in transcriptional complexes. For example, a combination of structural studies and photocrosslinking experiments with bacterial RNA polymerase in complex with Gfh1, an inhibitor of transcription initiation and elongation, revealed that a coiled-coil domain of Gfh1 blocks the NTP entry channel and freezes the RNA polymerase in an unusual ratcheted conformation (Tagami et al., 2010).

Two further studies extensively used **1** to study the topology of the RNA polymerase II transcription pre-initiation complex (PIC). Inserting **1** at multiple sites within the central cleft of RNA polymerase formed by the two large subunits Rpb1 and Rpb2 revealed differential interactions of the two subunits with the general transcription factors TFIIB, TFIIF, and TFIIE (Chen et al., 2007). Moreover, a detailed study based on local footprinting using the cysteine-reactive, hydroxyl radical-generating probe *p*-bromoacetamidobenzyl-EDTA-Fe(III) (Fe-BABE) and photocrosslinking with **1** provided insights into the structural arrangement and conformational dynamics of individual subunits of the general transcription factors TFIIE, TFIIH and TFIIB at the central cleft of RNA polymerase II within the PIC (Grunberg et al., 2012). Specifically, **1** was used to map the interaction surface between the TFIIE subunit Tfa2 and Ssl2, a subunit of TFIIH that contains RecA-like domains and is required for DNA opening and transition from the PIC to the open complex. **1** was introduced at several surface-exposed sites of the Ssl2 RecA-like domains and exhibited strong crosslinking tendency to Tfa2 (Figure 2B). Conversely, a number of positions bearing **1** unexpectedly showed crosslinking to TFIIB, which contradicted the current PIC model. In this model, these positions were in a distance of >30 Å from TFIIB, suggesting the existence of a second conformation of TFIIB. Taken together, these studies demonstrate the broad applicability of **1** to study protein interactions both *in vitro* and *in vivo*.

Recently, a novel photocrosslinking chemistry based on furan had been genetically encoded that offers complementary properties compared to the previously used chemistries (Figure 2C) (Schmidt and Summerer, 2013). In contrast to direct UV-light activation, this chemistry is indirectly activated by the *in situ* generation of singlet oxygen (^1^O_2_) and subsequent oxidation of the genetically encoded, furan-containing ncAA **2** (Scheme 1). Since ^1^O_2_ can be generated by irradiation of photosensitizers with red light, this approach should offer a high penetration depth in complex biological samples and the absence of nucleic acid damaging photoreactions associated with UV light. This chemistry had previously been described and thoroughly characterized in the context of DNA-DNA interstrand crosslink formation and proceeds via a proposed mechanism involving a 2+4 cycloaddition of ^1^O_2_ to the furan, opening of the resulting ozonide, and ultimately formation of a γ-keto-enal that can build cyclic adducts with A, G, and C nucleobases (Figure 2C) (Op De Beeck and Madder, 2011, 2012). Since this represents a rather nucleic acid-selective crosslinking chemistry that targets only certain nucleobases, it could provide more detailed information on the topology of protein-nucleic acid complexes, potentially including information about the binding mode of protein motifs (backbone- or groove-interaction) and the pairing state of individual nucleobases in complexed RNA or DNA. This chemistry was used to map the interaction of the HIV-1 transactivator of transcription (TAT) and its trans-activating response element (TAR), an interaction that is ubiquitous to HIV-1 mRNA transcription (Figure 2C) (Schmidt and Summerer, 2013). NcAA **2** was introduced at positions within the TAR-binding, arginine-rich motif of TAT and reported distinct orientations of the positions by differential crosslinking efficiencies.

### Photoactivation

A second widely employed application of ncAA has been the use of photocaged canonical amino acids to control the function of a protein with high spatiotemporal resolution. This approach is applicable for single amino acid functions of a protein that can be masked by photocaging or if the photocaging group itself can be positioned in a way that it perturbs the function of nearby amino acids/motifs, e.g., involved in catalytic activities or ligand recognition (Riggsbee and Deiters, 2010). Generally, photocaged versions of cysteine, tyrosine, serine and lysine have previously been genetically encoded, initially using *o*-nitrobenzyl groups that can be decaged with long-wave UV light (365 nm) (Liu and Schultz, 2010). Derivatives with improved photophysical and chemical properties were employed later, such as 4,5-dimethoxy-2-nitrobenzyl- or 4,5-dioxymethylene-2-nitrobenzyl- groups (Lemke et al., 2007; Gautier et al., 2010). These provide a bathochromic shift in absorption and enable the use of non-UV light for decaging. Additionally, alkylation of the benzylic methylene group has been a strategy to avoid the formation of reactive aldehydes as products of the photolysis reaction, thus further increasing the bio-orthogonality of the approach.

#### Transcription factors

In the generally first biological study that utilized ncAA in live eukaryotes, photocaged serine **3** (Scheme 1) was used to study a regulatory transport mechanism of the *S. cerevisiae* transcription factor Pho4 (Lemke et al., 2007). This ncAA was encoded in *S. cerevisiae* using an evolved LeuRS mutant of an *E. coli* tRNA^Leu^/LeuRS pair. Pho4 activates the expression of a number of genes in response to phosphate starvation. On the contrary in phosphate-rich conditions, Pho4 is phosphorylated at several serine sites by the cyclin-cyclin–dependent kinase complex Pho80/Pho85 and subsequently exported to the cytoplasm as a mechanism to downregulate its transcriptional activity (Komeili and O'Shea, 1999). In a Pho4-GFP fusion construct that exhibited regular transport behavior and activity, several critical serines were replaced by **3**, resulting in non-phosphorylated Pho4, even when cells were grown in phosphate-rich conditions. This was previously also observed in studies employing alanine mutants of the individual serines, which allowed to dissect their individual roles in nuclear export (Komeili and O'Shea, 1999). However, alanine mutants could only provide a static view on this highly dynamic process. In contrast, decaging of **3** by a millisecond laser pulse at 405 nm enabled phosphorylation and nuclear export with high spatiotemporal resolution. This allowed the direct, quantitative tracking of nuclear export and revealed distinct roles of the individual serines by differential export kinetics.

A second study extended the use of photocaged ncAA to mammalian cells, using a complementing approach to control the localization of a transcription factor. Photocaged lysine **5** (Scheme 1) was genetically encoded using an evolved *M. barkeri* PylRS mutant and used to mask a single lysine position in the nuclear localization sequence (NLS) of a fusion construct of the tumor suppressor p53 and GFP (Gautier et al., 2010; Lemke, 2010). In contrast to the wild type construct showing nuclear localization, the caged construct exhibited the same phenotype as a corresponding alanine mutant and was localized in the cytoplasm. Irradiation with a 5 s light pulse at 365 nm triggered nuclear import as result of decaging and restored a functional NLS.

#### RNA polymerase

In two pioneering studies of the Deiters group, photocaged ncAA have also been used to directly control the activity of RNA polymerases. In two examples, bacteriophage T7 RNA polymerase—that exhibits orthogonality in a broad range of prokaryotic and eukaryotic organisms—could be inactivated by introducing a single photocaged tyrosine **4** or lysine **5** (Scheme 1). ncAA **4** was genetically encoded in *E. coli* using a TyrRS mutant of the *M. jannschii* tRNA^Tyr^/TyrRS pair (Deiters et al., 2006) and later in eukaryotes using a PylRS mutant of the *M. barkeri* tRNA^Pyl^/PylRS pair (Arbely et al., 2012). Inactivation by caged ncAA **4** was achieved by substituting an active site tyrosine that plays an essential role in NTP-induced transition from the open to the closed conformation of T7 RNA polymerase during the catalytic cycle of the elongation state (Chou et al., 2010). Decaging resulted in restoration of catalytic activity both *in vitro* as well as in *E. coli* and HEK 293T cells indicated by reporter gene expressions, though the absence of orthogonality of the employed *M. jannaschii* TyrRS/tRNA^Tyr^ pair in eukaryotes required the transfection of T7 RNA polymerase protein in the latter case.

**Figure 3:**
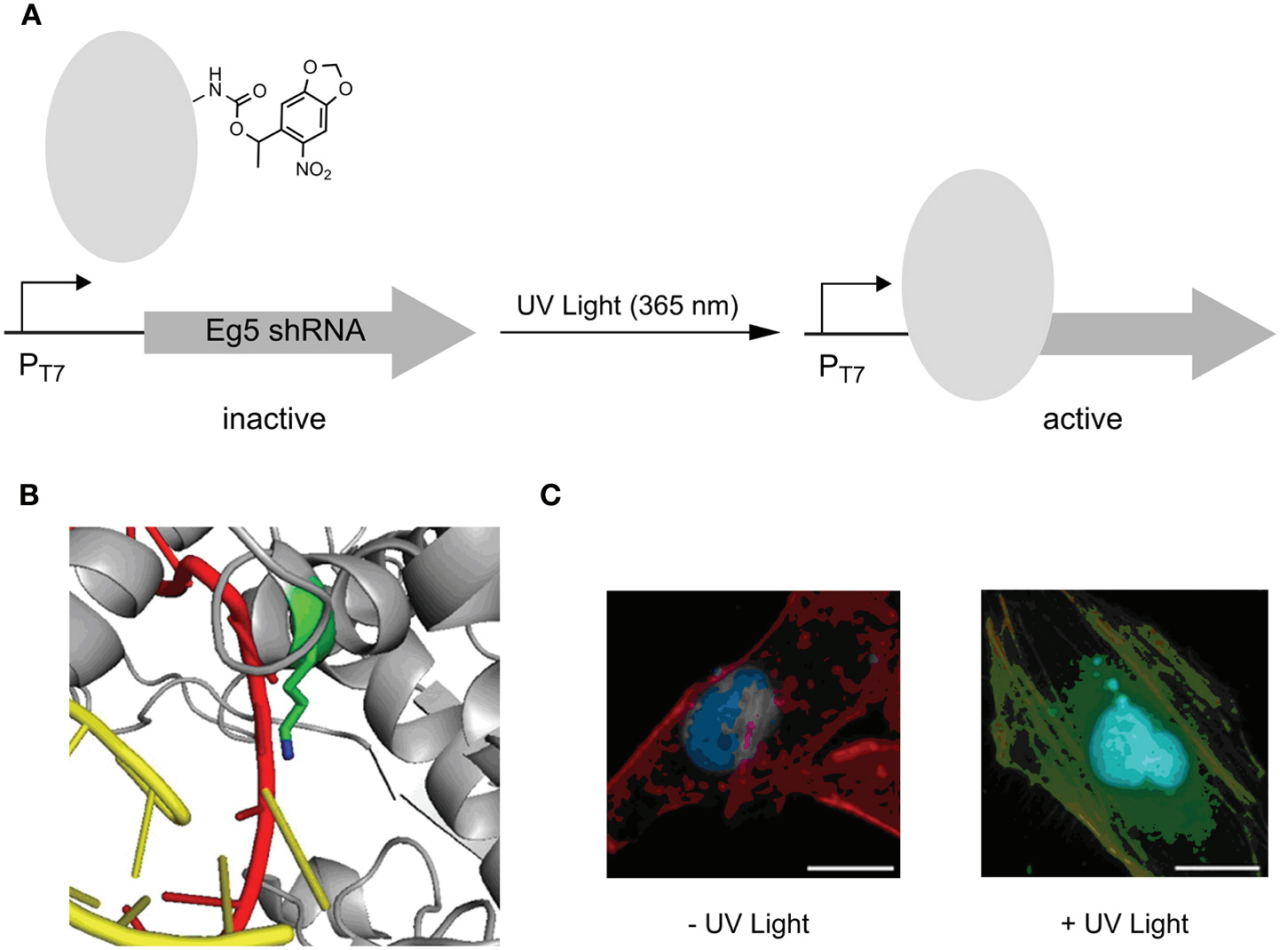
**Photoactivation of T7 RNA polymerase using photocaged lysine 5**. **(A)** General principle of activation of transcription of an anti-Eg5 shRNA. **(B)** Position of lysine used for replacement with 5, resulting in an inactive T7 RNA polymerase. **(C)** Photoactivation of trancription of anti-Eg5 shRNA and subsequent RNA_i_ -knockout of Eg5, resulting in a binuclear phenotype. Adapted with permission from Hemphill et al. (2013). Copyright (2013) American Chemical Society.

Very recently, photocaged lysine **5** was employed to control T7 RNA polymerase activity in an advanced setup (Figure 3A) (Hemphill et al., 2013). Since this ncAA is a substrate of the *M. barkeri* PylRS/tRNA^Pyl^ pair that exhibits orthogonality in all domains of life, the approach enabled the direct, intracellular expression of the photocaged protein. An active site lysine that is critical for catalytic activity because of its ability to recognize via the α-phosphate the incoming NTPs, was replaced by **5** (Figure 3B). This rendered the RNA polymerase completely inactive in mammalian reporter gene expressions until activation by irradiation at 365 nm. The potential of this approach was demonstrated by controlling the expression of a shRNA used for a knock-out of the Eg5 gene *via* RNA interference. Eg5 encodes a motor protein essential in mitosis as its results in monopolar spindles and mitotic arrest. This phenotype was successfully induced by UV light using the described caging strategy (Figure 3C).

### Posttranslational modifications

Posttranslational modifications greatly expand the chemical potential of the proteome and control various critical functions of proteins involved in transcription (Walsh et al., 2005). However, gaining insights into the precise roles of individual PTMs have been hampered by the difficulties to isolate or prepare selectively and homogenously modified proteins. These difficulties include the reversible and thus often incomplete modification of intracellular proteins, the presence of multiple modification sites and types and the challenges to separate the modified from non-modified proteins owing to the fact that often PTMs only promote subtle changes in the biophysical properties of the target protein (Latham and Dent, 2007). Consequently, there is a need to develop methods for the synthesis of homogenous proteins with defined PTMs in sufficient amounts for biochemical studies. This has started to be addressed by the direct genetic encoding of a variety of modified lysine with critical roles for both histones and transcription factors in studies by the groups of Chin, Liu, and Schultz (Liu et al., 2011).

#### Lysine methylation in histones

The reversible Nε-methylation of specific lysine residues in histones (Allfrey et al., 1964) is an important regulatory PTM that controls heterochromatin remodeling (Cheung and Lau, 2005). Nε-methylation can lead to differential methylation degrees with distinct functional roles, i.e. mono-, di-, and trimethylation (Taverna et al., 2007), and is orchestrated by histone methylases and demethylases.

A biochemical method to study methylation *in vitro* and in living cells by producing site-specifically Nε-methylated histones remained elusive for a long time. Methyltransferases have been used, but this approach is hampered by a limited control of regioselectivity and methylation extent, and for many sites, the respective methyltransferases have yet to be discovered (Martino et al., 2009). In contrast, advanced *in vitro* techniques, such as native chemical ligation (He et al., 2003; Chatterjee and Muir, 2010) or sortase-mediated ligation (Piotukh et al., 2011) have succeeded in producing homogeneously methylated histones.

**Scheme 2:**
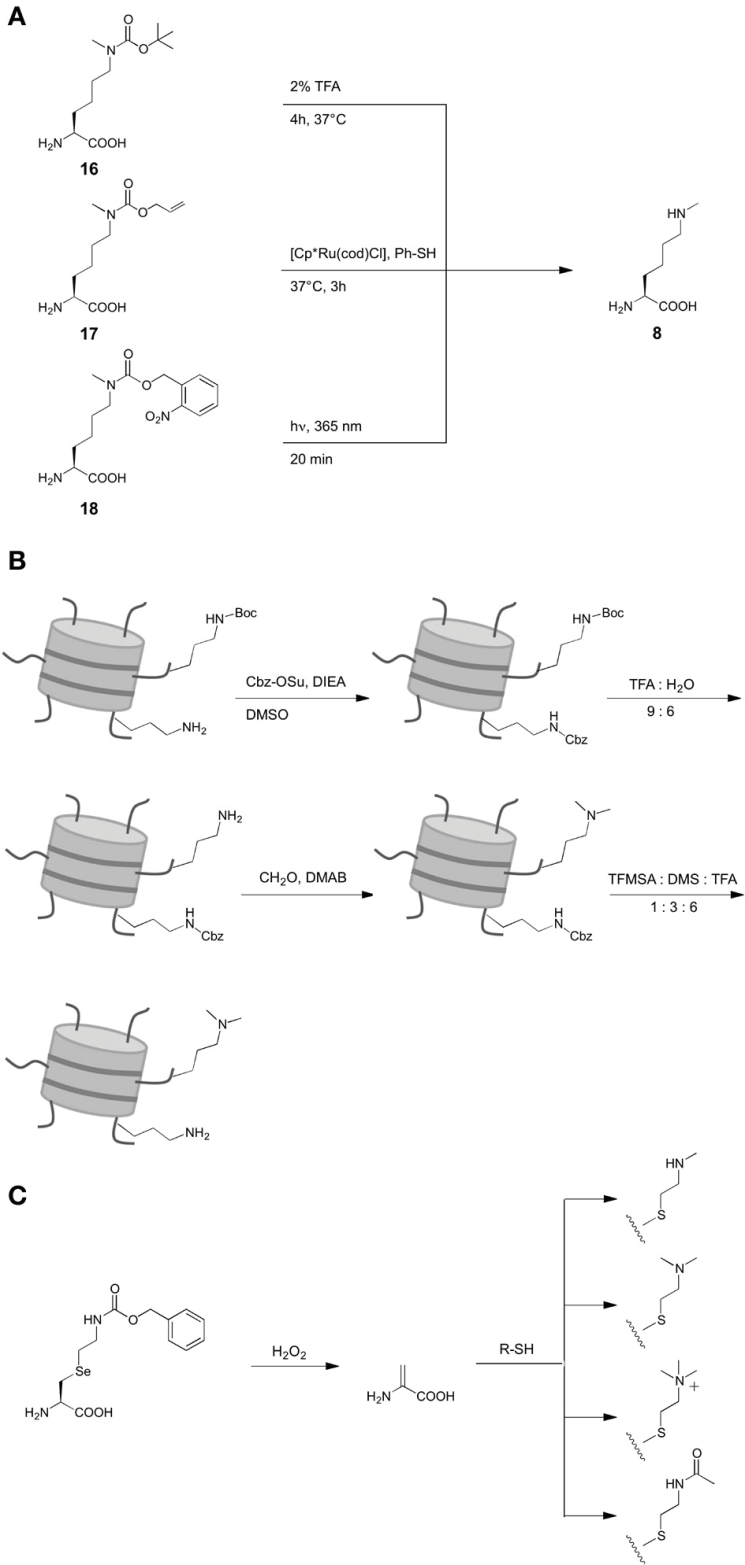
**Synthetic strategies for the introduction of different lysine PTM via genetic code expansion**. **(A)** Introduction of Nε-methyl-L-lysine. **(B)** Introduction of Nε-, Nε-dimethyl-L-lysine. **(C)** Flexible introduction of various PTM in form of L-lysine mimicks.

Only recently, the site-specific incorporation of the mono-methylated lysine Nε-methyl-L-lysine **6** (Scheme 1) was achieved by means of an expanded genetic code. However, presumably due to the structural similarity of lysine and **6**, no orthogonal aaRS could be evolved for this ncAA to date. Following an alternative strategy, Chin and coworkers (Nguyen et al., 2009) were able to genetically encode Nε-Boc-Nε-methyl-L-lysine **16** as precursor by employing the wild type of *M. barkeri* pyrrolysyl-tRNA synthetase. This ncAA could be incorporated into position K9 of histone H3, subsequently deprotected with 2% TFA and refolded (Scheme 2A). A related strategy that did not require denaturing and deprotection conditions was later introduced, based on Nε-Allyloxycarbonyl-Nε-methyl-L-lysine **17** (Scheme 2A) (Ai et al., 2010). This ncAA could be incorporated into position K27 of histone H2B using a Y384F mutant of *M. barkeri* PylRS and deprotected with the ruthenium catalyst [Cp^*^Ru(cod)Cl] under mild conditions in an aqueous environment. As another advancement offering increased bio-orthogonality and spatiotemporal control of deprotection, Schultz and Liu reported the genetic encoding of the photocaged ncAA Nε-(*o*-nitrobenzylcarbamoyl)-Nε-methyl-L-lysine **18** using evolved PylRS mutants (Scheme 2A) (Groff et al., 2010; Wang et al., 2010b). This ncAA can be deprotected by irradiation with UV light at 360 nm under mild conditions, which was demonstrated in mammalian cells (Groff et al., 2010).

In contrast to the various approaches to obtain mono-methylated lysine in histones, only one strategy for the genetic encoding of Nε, Nε-dimethyl-L-lysine **7** (Scheme 1) has been reported. No direct design of aaRS mutants capable of recognizing **7** or protected derivatives as substrates have been developed. Instead, posttranslational dimethylation in combination with two orthogonal protection groups was used in this case. First, Nε-Boc-L-lysine was incorporated at the target site K9 in histone H3 using wild type *M. barkeri* PylRS (Scheme 2B). In this mutant, all canonical lysine residues were protected with N-(benzyloxycarbonyloxy)succinimide (Cbz-OSu) under denaturing conditions. Subsequently, **7** was selectively deprotected with TFA: water (9: 6) at 4°C and dimethylated by reductive methylation using formaldehyde and dimethylaminoborane (DMAB). Finally, Cbz groups were removed selectively, yielding histone H3 with **7** at position K9 in natural conformation, as indicated by blots using anti-H3K9me2 and anti-H3K9me1 antibodies and co-immunoprecipitation experiments with heterochromatin protein 1 (HP1).

Though strategies have been developed for the introduction of both **6** and **7**, Nε, Nε, Nε-trimethyl-L-lysine has not yet been reported. However, a strategy to introduce amino acid modifications via selenocystein-derivatives proposed by Schultz (Wang et al., 2007a; Guo et al., 2008) allowed the incorporation of the trimethylated lysine mimick **12** (Scheme 1) into histone H3 (Wang et al., 2012). Initially described for phenyl-L-selenocysteine encoded by a *M. jannaschii* mutant and later for the derivative **19** (Scheme 2C) encoded by a *M. mazei* PylRS mutant, this approach is based on an oxidative elimination that results in dehydroalanine **20**. This ncAA can be derivatized by Michael addition reactions using thiols, resulting in a whole range of lysine PTM mimicks (**10–13**, (Schemes 1), 2C). Drawbacks of this methodology are the oxidative conditions of the reaction that can affect cysteines and methionines, the changes in the flexibility of the lysine linker and the pK_a_ of the Nε-ammonium group caused by the sulfur atom. However, the first drawback is not relevant for proteins with cysteines and methionines that can be mutated without loss of function and the approach offers considerable flexibility.

#### Lysine acetylation in histones

Nε-acetylation of lysine in histones is a reversible PTM that is catalyzed by histone acetylases and deacetylases and is critically involved in chromatin remodeling and thus transcriptional control (Jenuwein and Allis, 2001; Kouzarides, 2007). Besides the introduction of Nε-acetyl-L-lysine **8** (Scheme 1) by native chemical ligation (Shogren-Knaak et al., 2006), direct insertion by genetic encoding has been reported using an evolved *M. barkeri* PylRS mutant by Chin and coworkers (Neumann et al., 2008) that was further employed for the multiple incorporation of **8** into proteins (Huang et al., 2010). This ncAA was introduced (among other positions) into position K56 of histone H3 and used for FRET (fluorescence resonance energy transfer) studies on reconstituted nucleosomes. These revealed that acetylation of K56 does not have a direct, measurable effect on nucleosome stability and only moderately affects the activity of the nucleosome remodeling complexes Swi/Snf and RSC. However, DNA breathing (i.e. thermal motions that induce spontaneous openings and re-closings of the double helix) that can lead to unwrapping of DNA from the nucleosome, was increased by acetylation of K56 (Neumann et al., 2009).

In another study, the impact of acetylation of K16 in histone 4 on binding of Sir (silent information regulatory) proteins and linker DNA accessibility was investigated. This revealed that acetylation of K16 decreases the affinity of Sir3 for chromatin and affects chromatin structure. In contrast, the Sir2-4 subcomplex exhibited increased affinity when K16 was acetylated, suggesting a dual role of K16 acetylation, i.e. the recruitment of Sir2-4 and the repelling Sir3 (Oppikofer et al., 2011).

#### Lysine crotonylation in histones

Recent studies have revealed several acyl-groups, including crotonyl-, malonyl-, and succinyl-groups as novel lysine PTMs of histones (Du et al., 2011; Tan et al., 2011; Zhang et al., 2011b). Nε-Crotonyl-L-lysine **9** (Scheme 1) is mainly found in transcriptionally active chromatin and differs from Nε-acetyl-L-lysine in its regulation and genomic distribution (Tan et al., 2011). The genetic encoding of **9** was described by Schultz (Kim et al., 2012) and later by other groups using PylRS mutants (Gattner et al., 2013; Lee et al., 2013). This ncAA was incorporated into position K11 of human histone H2B and was recognized by an anti-Nε-crotonyl-L-lysine antibody (Kim et al., 2012).

#### Lysine acetylation in transcription factors

Acetylation of lysine, besides its critical role as histone PTM, is a widely found PTM in many different classes of proteins, including transcription factors (Kim et al., 2006). In this direction, Nε-acetyl-L-lysine **8** was used to study the role of K120 (that is acetylated in response to DNA-damage) in the DNA-binding domain (DBD) of the tumor suppressor p53 (Arbely et al., 2011). In its non-modified form, p53 is not capable to selectively bind to specific response elements under physiologic salt concentrations *in vitro*, but rather exhibits random binding. However, upon acetylation of K120, binding becomes selective under these conditions. Moreover, both p53 and p53 acetylated at K120 preferentially forms homocomplexes with DNA in a 4:1 stoichiometry, respectively, rather than mixed complexes containing both p53 modification states. This suggests that both DBD forms prefer distinct quaternary structures, which corroborates previous findings that p53 binds to non-specific and specific DNA sequences in differential quaternary structures.

In a second study, the influence of Nε-L-lysine acetylation on the activity of transcription factors in *E. coli* was investigated (Thao et al., 2010). A proteome screen for substrates of the Gcn5-like protein acetyltransferase (Pat) afforded four transcription factors as substrates. One of them (RcsB) was found to be acetylated at position K180, moreover, it was found that this was a reversible process since K180 could also be deacetylated by CobB, a sirtuin-like protein deacetylase. Electromobility shift assays with RcsB bearing **8** revealed that K180 acetylation of RcsB was critical for DNA binding, suggesting for the first time that bacteria use this PTM to regulate gene expression.

### Other ncAA functions

Beside the three main applications of ncAA described above, a number of other ncAA functions have been used in the context of transcription factor-DNA complexes in earlier studies. Though these studies so far were isolated proof-of-concept experiments, the employed ncAA offer a significant potential to be used more widely in the field.

For example, the genetic encoding of the azobenzene-containing amino acid **14** (Scheme 1) offers properties for the photocontrol of protein functions that complement the use of photocaged amino acids (Bose et al., 2006). ncAA **14** can undergo a reversible cis-trans photoisomerization: irradiation at 320–340 nm converts the more stable *trans*- to the *cis*-isomer that can re-isomerize thermally or upon irradiation at ≥420 nm. Both isomers differ in geometry and dipole which can be exploited to photomodulate the structure and consequently the binding affinity and/or activity of a protein. This was demonstrated by photomodulating the affinity of *E. coli* CAP for cAMP and consequently the binding to its cognate DNA binding site in the lac promoter.

Another promising advancement has been the genetic encoding of the bipyridyl-containing ncAA **15** that can serve as a metal ion chelator (Xie et al., 2007). This ncAA, when chelating Fe(II) or Cu(II) in the presence of a reducing agent, can trigger the oxidative cleavage of the DNA backbone and thus it is a useful probe to map protein DNA interactions, though with limited potential for intracellular applications (Lee and Schultz, 2008).

Finally, dimerization events upon DNA-binding of a Gcn4 bZIP protein were monitored by quenching intrinsic tryptophan fluorescence in the protein using the ncAA *p*-nitro-L-phenylalanine (Tsao et al., 2006).

## Conclusions and outlook

The here reviewed studies demonstrate the considerable potential of genetic code expansion to provide detailed insights into the molecular mechanisms that underlie transcription and its regulation. These insights are as diverse as the chemical functions of the employed ncAA, and in many cases could not have been provided by traditional approaches of molecular biology. In respect to the main ncAA functions discussed here, several aspects for future improvements can be envisaged. Photocrosslinking ncAA could be developed with additional, complementary chemoselectivities that could provide an additional layer of information into the protein complexes under study, besides the sole vicinity of interaction partners usually provided by non-specific photocrosslinkers. Moreover, structural aspects of photocrosslinking ncAA can be considered: In view of high success-rates for the discovery of unknown interaction partners, wide-range reactivity of photocrosslinkers with long, flexible linkers is desirable (Chou et al., 2011; Zhang et al., 2011a; Yanagisawa et al., 2012; Schmidt and Summerer, 2013). However, the mapping of interaction surfaces of known complexes with increased resolution would benefit from small, rather rigid crosslinkers. In both cases, perturbation of binding has to be minimal. Though the applicability of both photocrosslinking and photoactivatable ncAA using UV-light for activation has been thoroughly proven, the use of red light with a wider range of such ncAA would open new perspectives for the studying of complex samples, such as multicellular organisms. Additionally, ncAA functions that are explicitly nucleic acid-directed (e.g., nucleolytic backbone cleavers) and are applicable *in vivo* could lead to interesting tools to study protein-nucleic acid interactions involved in transcription. Finally, since currently encodable PTMs represent only a subset of the ones found in nature, the genetic encoding of more PTMs would be highly attractive.

From such methodological advancements, in combination with the growing number of organisms with expandable genetic code, a multitude of insights into the biological functions of proteins in their native, intracellular environment can be expected.

### Conflict of interest statement

The authors declare that the research was conducted in the absence of any commercial or financial relationships that could be construed as a potential conflict of interest.

## References

[B1] Ai H. W. Lee J. W. Schultz P. G. (2010). A method to site-specifically introduce methyllysine into proteins in E. coli. Chem. Commun. 46, 5506–5508 10.1039/C0cc00108b 20571694 PMC2928331

[B2] Ai H. W. Shen W. Sagi A. Chen P. R. Schultz P. G. (2011). Probing protein-protein interactions with a genetically encoded photo-crosslinking amino acid. Chembiochem 12, 1854–1857 10.1002/cbic.201100194 21678540

[B3] Allfrey V. G. Faulkner R. Mirsky A. E. (1964). Acetylation and methylation of histones and their possible role in the regulation of RNA synthesis. Proc. Natl. Acad. Sci. U.S.A. 51, 786–794 10.1073/pnas.51.5.786 14172992 PMC300163

[B4] Arbely E. Natan E. Brandt T. Allen M. D. Veprintsev D. B. Robinson C. V. (2011). Acetylation of lysine 120 of p53 endows DNA-binding specificity at effective physiological salt concentration. Proc. Natl. Acad. Sci. U.S.A. 108, 8251–8256 10.1073/pnas.1105028108 21525412 PMC3100949

[B5] Arbely E. Torres-Kolbus J. Deiters A. Chin J. W. (2012). Photocontrol of tyrosine phosphorylation in mammalian cells via genetic encoding of photocaged tyrosine. J. Am. Chem. Soc. 134, 11912–11915 10.1021/ja3046958 22758385

[B6] Bernstein B. E. Birney E. Dunham I. Green E. D. Gunter C. Snyder M. (2012). An integrated encyclopedia of DNA elements in the human genome. Nature 489, 57–74 10.1038/nature11247 22955616 PMC3439153

[B7] Bianco A. Townsley F. M. Greiss S. Lang K. Chin J. W. (2012). Expanding the genetic code of Drosophila melanogaster. Nat. Chem. Biol. 8, 748 10.1038/nchembio.1043 22864544

[B8] Bose M. Groff D. Xie J. Brustad E. Schultz P. G. (2006). The incorporation of a photoisomerizable amino acid into proteins in E. coli. J. Am. Chem. Soc. 128, 388–389 10.1021/ja055467u 16402807

[B9] Chatterjee A. Xiao H. Bollong M. Ai H. W. Schultz P. G. (2013). Efficient viral delivery system for unnatural amino acid mutagenesis in mammalian cells. Proc. Natl. Acad. Sci. U.S.A. 110, 11803–11808 10.1073/pnas.1309584110 23818609 PMC3718144

[B10] Chatterjee C. Muir T. W. (2010). Chemical approaches for studying histone modifications. J. Biol. Chem. 285, 11045–11050 10.1074/jbc.R109.080291 20147749 PMC2856977

[B11] Chen H. T. Warfield L. Hahn S. (2007). The positions of TFIIF and TFIIE in the RNA polymerase II transcription preinitiation complex. Nat. Struct. Mol. Biol. 14, 696–703 10.1038/Nsmb1272 17632521 PMC2483787

[B12] Cheung P. Lau P. (2005). Epigenetic regulation by histone methylation and histone variants. Mol. Endocrinol. 19, 563–573 10.1210/me.2004-0496 15677708

[B13] Chin J. W. Cropp T. A. Anderson J. C. Mukherji M. Zhang Z. Schultz P. G. (2003). An expanded eukaryotic genetic code. Science 301, 964–967 10.1126/science.1084772 12920298

[B14] Chin J. W. Martin A. B. King D. S. Wang L. Schultz P. G. (2002a). Addition of a photocrosslinking amino acid to the genetic code of Escherichiacoli. Proc. Natl. Acad. Sci. U.S.A. 99, 11020–11024 10.1073/pnas.172226299 12154230 PMC123203

[B15] Chin J. W. Santoro S. W. Martin A. B. King D. S. Wang L. Schultz P. G. (2002b). Addition of p-azido-L-phenylalanine to the genetic code of *Escherichia coli*. J. Am. Chem. Soc. 124, 9026–9027 10.1021/ja027007w 12148987

[B16] Chou C. J. Uprety R. Davis L. Chin J. W. Deiters A. (2011). Genetically encoding an aliphatic diazirine for protein photocrosslinking. Chem. Sci. 2, 480–483 10.1039/C0sc00373e

[B17] Chou C. J. Young D. D. Deiters A. (2010). Photocaged T7 RNA polymerase for the light activation of transcription and gene function in pro- and Eukaryotic cells. Chembiochem 11, 972–977 10.1002/Cbic.201000041 20301166 PMC3762680

[B18] Deiters A. Groff D. Ryu Y. Xie J. Schultz P. G. (2006). A genetically encoded photocaged tyrosine. Angew. Chem. Int. Ed. Engl. 45, 2728–2731 10.1002/anie.200600264 16548032

[B19] Djebali S. Davis C. A. Merkel A. Dobin A. Lassmann T. Mortazavi A. (2012). Landscape of transcription in human cells. Nature 489, 101–108 10.1038/nature11233 22955620 PMC3684276

[B20] Du J. Zhou Y. Su X. Yu J. J. Khan S. Jiang H. (2011). Sirt5 is a NAD-dependent protein lysine demalonylase and desuccinylase. Science 334, 806–809 10.1126/science.1207861 22076378 PMC3217313

[B21] Fuxreiter M. Tompa P. Simon I. Uversky V. N. Hansen J. C. Asturias F. J. (2008). Malleable machines take shape in eukaryotic transcriptional regulation. Nat. Chem. Biol. 4, 728–737 10.1038/Nchembio.127 19008886 PMC2921704

[B22] Galardy R. E. Craig L. C. Printz M. P. (1973). Benzophenone triplet - new photochemical probe of biological ligand-receptor interactions. Nat. New Biol. 242, 127–128 10.1038/newbio242127a0 4513418

[B23] Gattner M. J. Vrabel M. Carell T. (2013). Synthesis of epsilon-N-propionyl-, epsilon-N-butyryl-, and epsilon-N-crotonyl-lysine containing histone H3 using the pyrrolysine system. Chem. Commun. (Camb.) 49, 379–381 10.1039/c2cc37836a 23192406

[B24] Gautier A. Nguyen D. P. Lusic H. An W. A. Deiters A. Chin J. W. (2010). Genetically encoded photocontrol of protein localization in mammalian cells. J. Am. Chem. Soc. 132, 4086 10.1021/Ja910688s 20218600

[B25] Greiss S. Chin J. W. (2011). Expanding the genetic code of an animal. J. Am. Chem. Soc. 133, 14196 10.1021/ja2054034 21819153 PMC3168933

[B26] Groff D. Chen P. R. Peters F. B. Schultz P. G. (2010). A genetically encoded ε-N-methyl lysine in mammalian cells. Chembiochem 11, 1066–1068 10.1002/cbic.200900690 20422671 PMC2882943

[B27] Grunberg S. Warfield L. Hahn S. (2012). Architecture of the RNA polymerase II preinitiation complex and mechanism of ATP-dependent promoter opening. Nat. Struct. Mol. Biol. 19, 788–796 10.1038/Nsmb.2334 22751016 PMC3414687

[B28] Guo J. Wang J. Lee J. S. Schultz P. G. (2008). Site-specific incorporation of methyl- and acetyl-lysine analogues into recombinant proteins. Angew. Chem. Int. Ed. Engl. 47, 6399–6401 10.1002/anie.200802336 18624319

[B29] He S. Bauman D. Davis J. S. Loyola A. Nishioka K. Gronlund J. L. (2003). Facile synthesis of site-specifically acetylated and methylated histone proteins: reagents for evaluation of the histone code hypothesis. Proc. Natl. Acad. Sci. U.S.A. 100, 12033–12038 10.1073/pnas.2035256100 14530408 PMC218708

[B30] Hemphill J. Chou C. Chin J. W. Deiters A. (2013). Genetically encoded light-activated transcription for spatiotemporal control of gene expression and gene silencing in mammalian cells. J. Am. Chem. Soc. 135, 13433–13439 10.1021/ja4051026 23931657 PMC4188981

[B31] Huang Y. Russell W. K. Wan W. Pai P. J. Russell D. H. Liu W. (2010). A convenient method for genetic incorporation of multiple noncanonical amino acids into one protein in Escherichia coli. Mol. Biosyst. 6, 683–686 10.1039/b920120c 20237646

[B32] Jenuwein T. Allis C. D. (2001). Translating the histone code. Science 293, 1074–1080 10.1126/science.1063127 11498575

[B33] Johnson D. B. Xu J. Shen Z. Takimoto J. K. Schultz M. D. Schmitz R. J. (2011). RF1 knockout allows ribosomal incorporation of unnatural amino acids at multiple sites. Nat. Chem. Biol. 7, 779 10.1038/nchembio.657 21926996 PMC3201715

[B34] Kang J. Y. Kawaguchi D. Coin I. Xiang Z. O'Leary D. D. M. Slesinger P. A. (2013). *In vivo* expression of a light-activatable potassium channel using unnatural amino acids. Neuron 80, 358–370 10.1016/J.Neuron.2013.08.016 24139041 PMC3815458

[B35] Kauer J. C. Ericksonviitanen S. Wolfe H. R. Degrado W. F. (1986). Para-benzoyl-L-phenylalanine, a new photoreactive amino-acid - photolabeling of calmodulin with a synthetic calmodulin-binding peptide. J. Biol. Chem. 261, 695–700 3733726

[B36] Kim C. H. Kang M. Kim H. J. Chatterjee A. Schultz P. G. (2012). Site-specific incorporation of epsilon-N-crotonyllysine into histones. Angew. Chem. Int. Ed. Engl. 51, 7246–7249 10.1002/Anie.201203349 22689270 PMC3783207

[B37] Kim S. C. Sprung R. Chen Y. Xu Y. Ball H. Pei J. (2006). Substrate and functional diversity of lysine acetylation revealed by a proteomics survey. Mol. Cell 23, 607–618 10.1016/j.molcel.2006.06.026 16916647

[B38] Komeili A. O'Shea E. K. (1999). Roles of phosphorylation sites in regulating activity of the transcription factor Pho4. Science 284, 977–980 10.1126/Science.284.5416.977 10320381

[B39] Kouzarides T. (2007). Chromatin modifications and their function. Cell 128, 693–705 10.1016/J.Cell.2007.02.005 17320507

[B40] Krishnamurthy M. Dugan A. Nwokoye A. Fung Y. H. Lancia J. K. Majmudar C. Y. (2011). Caught in the act: covalent cross-linking captures activator-coactivator interactions *in vivo*. ACS Chem. Biol. 6, 1321–1326 10.1021/Cb200308e 21977905 PMC3245988

[B41] Lacey V. K. Louie G. V. Noel J. P. Wang L. (2013). Expanding the library and substrate diversity of the pyrrolysyl-tRNA synthetase to incorporate unnatural amino acids containing conjugated rings. Chembiochem. 14, 2100–2105 10.1002/cbic.201300400 24019075 PMC3947478

[B42] Lajoie M. J. Rovner A. J. Goodman D. B. Aerni H. R. Haimovich A. D. Kuznetsov G. (2013). Genomically recoded organisms expand biological functions. Science 342, 357–360 10.1126/Science.1241459 24136966 PMC4924538

[B43] Latham J. A. Dent S. Y. (2007). Cross-regulation of histone modifications. Nat. Struct. Mol. Biol. 14, 1017–1024 10.1038/nsmb1307 17984964

[B44] Lee H. S. Dimla R. D. Schultz P. G. (2009). Protein-DNA photo-crosslinking with a genetically encoded benzophenone-containing amino acid. Bioorg. Med. Chem. Lett. 19, 5222–5224 10.1016/j.bmcl.2009.07.011 19643606 PMC2873854

[B45] Lee H. S. Schultz P. G. (2008). Biosynthesis of a site-specific DNA cleaving protein. J. Am. Chem. Soc. 130, 13194–13195 10.1021/ja804653f 18788806

[B46] Lee Y. J. Wu B. Raymond J. E. Zeng Y. Fang X. Wooley K. L. (2013). A genetically encoded acrylamide functionality. ACS Chem. Biol. 8, 1664–1670 10.1021/cb400267m 23735044 PMC3746000

[B47] Lemke E. A. (2010). Precision control of cellular pathways with light. Chembiochem 11, 1825–1827 10.1002/Cbic.201000364 20687052

[B48] Lemke E. A. Summerer D. Geierstanger B. H. Brittain S. M. Schultz P. G. (2007). Control of protein phosphorylation with a genetically encoded photocaged amino acid. Nat. Chem. Biol. 3, 769–772 10.1038/nchembio.2007.44 17965709

[B49] Li F. Zhang H. Sun Y. Pan Y. Zhou J. Wang J. (2013). Expanding the genetic code for photoclick chemistry in *E. coli*, mammalian cells, and *A. thaliana. Angew.* Chem. Int. Ed. Engl. 52, 9700–9704 10.1002/anie.201303477 23873613

[B50] Lin S. X. Zhang Z. R. Xu H. Li L. Chen S. Li J. (2011). Site-specific incorporation of photo-cross-linker and bioorthogonal amino acids into enteric bacterial pathogens. J. Am. Chem. Soc. 133, 20581–20587 10.1021/Ja209008w 22084898

[B51] Liu C. C. Schultz P. G. (2010). Adding new chemistries to the genetic code. Ann. Rev. Biochem. 79, 413 10.1146/Annurev.Biochem.052308.105824 20307192

[B52] Liu W. S. R. Wang Y. S. Wan W. (2011). Synthesis of proteins with defined posttranslational modifications using the genetic noncanonical amino acid incorporation approach. Mol. Biosyst. 7, 38–47 10.1039/C0mb00216j 21088799

[B53] Majmudar C. Y. Lee L. W. Lancia J. K. Nwokoye A. Wang Q. Wands A. M. (2009a). Impact of nonnatural amino acid mutagenesis on the *in vivo* function and binding modes of a transcriptional activator. J. Am. Chem. Soc. 131, 14240–14242 10.1021/Ja904378z 19764747 PMC4182099

[B54] Majmudar C. Y. Wang B. Lum J. K. Hakansson K. Mapp A. K. (2009b). A High-resolution interaction map of three transcriptional activation domains with a key coactivator from photo-cross-linking and multiplexed mass spectrometry. Angew. Chem. Int. Ed. Engl. 48, 7021–7024 10.1002/Anie.200902669 19681084 PMC3222623

[B55] Mapp A. K. Ansari A. Z. (2007). A TAD further: exogenous control of gene activation. ACS Chem. Biol. 2, 62–75 10.1021/Cb600463w 17243784

[B56] Martino F. Kueng S. Robinson P. Tsai-Pflugfelder M. Van Leeuwen F. Ziegler M. (2009). Reconstitution of yeast silent chromatin: multiple contact sites and O-AADPR binding load SIR complexes onto nucleosomes *in vitro*. Mol. Cell 33, 323–334 10.1016/J.Molcel.2009.01.009 19217406

[B57] Melcher K. (2000). The strength of acidic activation domains correlates with their affinity for both transcriptional and non-transcriptional proteins. J. Mol. Biol. 301, 1097–1112 10.1006/Jmbi.2000.4034 10966808

[B58] Mohibullah N. Hahn S. (2008). Site-specific cross-linking of TBP *in vivo* and *in vitro* reveals a direct functional interaction with the SAGA subunit Spt3. Genes Dev. 22, 2994–3006 10.1101/Gad.1724408 18981477 PMC2577793

[B59] Mukai T. Hayashi A. Iraha F. Sato A. Ohtake K. Yokoyama S. (2010). Codon reassignment in the Escherichia coli genetic code. Nucleic Acids Res. 38, 8188–8195 10.1093/nar/gkq707 20702426 PMC3001078

[B60] Munzel M. Globisch D. Carell T. (2011). 5-Hydroxymethylcytosine, the sixth base of the genome. Angew. Chem. Int. Ed Engl. 50, 6460–6468 10.1002/anie.201101547 21688365

[B61] Neph S. Vierstra J. Stergachis A. B. Reynolds A. P. Haugen E. Vernot B. (2012). An expansive human regulatory lexicon encoded in transcription factor footprints. Nature 489, 83–90 10.1038/nature11212 22955618 PMC3736582

[B62] Neumann H. Hancock S. M. Buning R. Routh A. Chapman L. Somers J. (2009). A method for genetically installing site-specific acetylation in recombinant histones defines the effects of H3 K56 acetylation. Mol. Cell 36, 153–163 10.1016/j.molcel.2009.07.027 19818718 PMC2856916

[B63] Neumann H. Peak-Chew S. Y. Chin J. W. (2008). Genetically encoding N-epsilon-acetyllysine in recombinant proteins. Nat. Chem. Biol. 4, 232–234 10.1038/nchembio.73 18278036

[B64] Neumann H. Wang K. Davis L. Garcia-Alai M. Chin J. W. (2010). Encoding multiple unnatural amino acids via evolution of a quadruplet-decoding ribosome. Nature 464, 441–444 10.1038/nature08817 20154731

[B65] Nguyen D. P. Garcia Alai M. M. Kapadnis P. B. Neumann H. Chin J. W. (2009). Genetically encoding Nε-methyl-l-lysine in recombinant histones. J. Am. Chem. Soc. 131, 14194–14195 10.1021/ja906603s 19772323

[B66] Olsen C. A. (2012). Expansion of the lysine acylation landscape. Angew. Chem. Int. Ed. Engl. 51, 3755–3756 10.1002/Anie.201200316 22374739

[B67] Op De Beeck M. Madder A. (2011). Unprecedented C-selective interstrand cross-linking through *in situ* oxidation of furan-modified oligodeoxynucleotides. J. Am. Chem. Soc. 133, 796–807 10.1021/Ja1048169 21162525

[B68] Op De Beeck M. Madder A. (2012). Sequence specific DNA cross-linking triggered by visible light. J. Am. Chem. Soc. 134, 10737–10740 10.1021/Ja301901p 22698383

[B69] Oppikofer M. Kueng S. Martino F. Soeroes S. Hancock S. M. Chin J. W. (2011). A dual role of H4K16 acetylation in the establishment of yeast silent chromatin. EMBO J. 30, 2610–2621 10.1038/emboj.2011.170 21666601 PMC3155304

[B70] Parrish A. R. She X. Xiang Z. Coin I. Shen Z. Briggs S. P. (2012). Expanding the genetic code of Caenorhabditis elegans using bacterial aminoacyl-tRNA synthetase/tRNA pairs. ACS Chem. Biol. 7, 1292 10.1021/cb200542j 22554080 PMC3401359

[B71] Piotukh K. Geltinger B. Heinrich N. Gerth F. Beyermann M. Freund C. (2011). Directed evolution of sortase A mutants with altered substrate selectivity profiles. J. Am. Chem. Soc. 133, 17536–17539 10.1021/ja205630g 21978125

[B72] Riggsbee C. W. Deiters A. (2010). Recent advances in the photochemical control of protein function. Trends Biotechnol. 28, 468–475 10.1016/j.tibtech.2010.06.001 20667607 PMC2926219

[B73] Ryden S. M. Isaksson L. A. (1984). A temperature-sensitive mutant of Escherichia-coli that shows enhanced misreading of UAG/A and increased efficiency for some transfer-RNA nonsense suppressors. Mol. Gen. Genet. 193, 38–45 10.1007/Bf00327411 6419024

[B74] Schmidt M. J. Summerer D. (2013). Red-light-controlled protein-RNA crosslinking with a genetically encoded furan. Angew. Chem. Int. Ed. Engl. 52, 4690 10.1002/anie.201300754 23512703

[B75] Shogren-Knaak M. Ishii H. Sun J. M. Pazin M. J. Davie J. R. Peterson C. L. (2006). Histone H4-K16 acetylation controls chromatin structure and protein interactions. Science 311, 844–847 10.1126/Science.1124000 16469925

[B76] Song C. X. Yi C. Q. He C. (2012). Mapping recently identified nucleotide variants in the genome and transcriptome. Nat. Biotechnol. 30, 1107–1116 10.1038/Nbt.2398 23138310 PMC3537840

[B77] Tagami S. Sekine S. Kumarevel T. Hino N. Murayama Y. Kamegamori S. (2010). Crystal structure of bacterial RNA polymerase bound with a transcription inhibitor protein. Nature 468, 978–982 10.1038/nature09573 21124318

[B78] Tan M. Luo H. Lee S. Jin F. Yang J. S. Montellier E. (2011). Identification of 67 histone marks and histone lysine crotonylation as a new type of histone modification. Cell 146, 1016–1028 10.1016/j.cell.2011.08.008 21925322 PMC3176443

[B79] Taverna S. D. Li H. Ruthenburg A. J. Allis C. D. Patel D. J. (2007). How chromatin-binding modules interpret histone modifications: lessons from professional pocket pickers. Nat. Struct. Mol. Biol. 14, 1025–1040 10.1038/nsmb1338 17984965 PMC4691843

[B80] Thao S. Chen C. S. Zhu H. Escalante-Semerena J. C. (2010). Nepsilon-lysine acetylation of a bacterial transcription factor inhibits Its DNA-binding activity. PLoS ONE 5:e15123 10.1371/journal.pone.0015123 21217812 PMC3013089

[B81] Thurman R. E. Rynes E. Humbert R. Vierstra J. Maurano M. T. Haugen E. (2012). The accessible chromatin landscape of the human genome. Nature 489, 75–82 10.1038/nature11232 22955617 PMC3721348

[B82] Tippmann E. M. Liu W. Summerer D. Mack A. V. Schultz P. G. (2007). A genetically encoded diazirine photocrosslinker in *Escherichia coli*. Chembiochem 8, 2210–2214 10.1002/cbic.200700460 18000916

[B83] Tsao M. L. Summerer D. Ryu Y. Schultz P. G. (2006). The genetic incorporation of a distance probe into proteins in *Escherichia coli*. J. Am. Chem. Soc. 128, 4572–4573 10.1021/ja058262u 16594684

[B84] Walsh C. T. Garneau-Tsodikova S. Gatto G. J. Jr. (2005). Protein posttranslational modifications: the chemistry of proteome diversifications. Angew. Chem. Int. Ed Engl. 44, 7342–7372 10.1002/anie.200501023 16267872

[B85] Wang F. Robbins S. Guo J. T. Shen W. J. Schultz P. G. (2010a). Genetic incorporation of unnatural amino acids into proteins in Mycobacterium tuberculosis. PLoS ONE 5:e9354 10.1371/journal.pone.0009354 20179771 PMC2825273

[B86] Wang J. Schiller S. M. Schultz P. G. (2007a). A biosynthetic route to dehydroalanine-containing proteins. Angew. Chem. Int. Ed Engl. 46, 6849–6851 10.1002/anie.200702305 17685371

[B87] Wang K. H. Neumann H. Peak-Chew S. Y. Chin J. W. (2007b). Evolved orthogonal ribosomes enhance the efficiency of synthetic genetic code expansion. Nat. Biotechnol. 25, 770–777 10.1038/Nbt1314 17592474

[B88] Wang Y. S. Wu B. Wang Z. Y. Huang Y. Wan W. Russell W. K. (2010b). A genetically encoded photocaged N-epsilon-methyl-L-lysine. Mol. Biosyst. 6, 1575–1578 10.1039/C002155e 20711534

[B89] Wang Z. Y. U. Wang Y. S. Pai P. J. Russell W. K. Russell D. H. Liu W. S. R. (2012). A facile method to synthesize histones with posttranslational modification mimics. Biochemistry 51, 5232–5234 10.1021/Bi300535a 22697363 PMC3448024

[B90] Wilson B. G. Roberts C. W. M. (2011). SWI/SNF nucleosome remodellers and cancer. Nat. Rev. Cancer 11, 481–492 10.1038/Nrc3068 21654818

[B91] Wu I. L. Patterson M. A. Desai H. E. C. Mehl R. A. Giorgi G. Conticello V. P. (2013). Multiple site-selective insertions of noncanonical amino acids into sequence-repetitive polypeptides. Chembiochem 14, 968–978 10.1002/Cbic.201300069 23625817 PMC3786561

[B92] Xie J. Liu W. Schultz P. G. (2007). A genetically encoded bidentate, metal-binding amino acid. Angew. Chem. Int. Ed Engl. 46, 9239–9242 10.1002/anie.200703397 17893898

[B93] Yanagisawa T. Hino N. Iraha F. Mukai T. Sakamoto K. Yokoyama S. (2012). Wide-range protein photo-crosslinking achieved by a genetically encoded N(epsilon)-(benzyloxycarbonyl)lysine derivative with a diazirinyl moiety. Mol. Biosyst. 8, 1131–1135 10.1039/c2mb05321g 22294092

[B94] Young T. S. Ahmad I. Brock A. Schultz P. G. (2009). Expanding the genetic repertoire of the methylotrophic yeast *Pichia pastoris*. Biochemistry 48, 2643–2653 10.1021/bi802178k 19265424

[B95] Zhang M. Lin S. Song X. Liu J. Fu Y. Ge X. (2011a). A genetically incorporated crosslinker reveals chaperone cooperation in acid resistance. Nat. Chem. Biol. 7, 671–677 10.1038/nchembio.644 21892184

[B96] Zhang Z. Tan M. Xie Z. Dai L. Chen Y. Zhao Y. (2011b). Identification of lysine succinylation as a new post-translational modification. Nat. Chem. Biol. 7, 58–63 10.1038/nchembio.495 21151122 PMC3065206

